# Generative models for sequential dynamics in active inference

**DOI:** 10.1007/s11571-023-09963-x

**Published:** 2023-04-26

**Authors:** Thomas Parr, Karl Friston, Giovanni Pezzulo

**Affiliations:** 1 Wellcome Centre for Human Neuroimaging, Queen Square Institute of Neurology, University College London, London, UK; 2 Institute of Cognitive Sciences and Technologies, National Research Council, Via S. Martino Della Battaglia, 44, 00185 Rome, Italy

**Keywords:** Bayesian, Active inference, Generative model, Variational, Sequential dynamics

## Abstract

A central theme of theoretical neurobiology is that most of our cognitive operations require processing of discrete sequences of items. This processing in turn emerges from continuous neuronal dynamics. Notable examples are sequences of words during linguistic communication or sequences of locations during navigation. In this perspective, we address the problem of sequential brain processing from the perspective of active inference, which inherits from a Helmholtzian view of the predictive (Bayesian) brain. Underneath the active inference lies a generative model; namely, a probabilistic description of how (observable) consequences are generated by (unobservable) causes. We show that one can account for many aspects of sequential brain processing by assuming the brain entails a generative model of the sensed world that comprises central pattern generators, narratives, or well-defined sequences. We provide examples in the domains of motor control (e.g., handwriting), perception (e.g., birdsong recognition) through to planning and understanding (e.g., language). The solutions to these problems include the use of sequences of attracting points to direct complex movements—and the move from continuous representations of auditory speech signals to the discrete words that generate those signals.

## Introduction

The dynamics of cognitive systems are often characterised in terms of sequences. These range from sequences of words in linguistic communication (Friston et al. 2020a) to the sequences of steps we might take while navigating a maze (Kaplan and Friston 2018). From a dynamical systems perspective, this implies a series of unstable fixed points that are visited in turn (Afraimovich et al. 2004). This idea is (implicitly) central to most neurobiological theories of planning and behaviour, and manifests in the notion of a *cognon* (Rabinovich et al. in preparation) or elementary cognitive unit. Simply put, the hypothesis is that cognition is underwritten by an emergence of discrete sequences of events from the continuous evolution of neural activity. This reflects a recent trend in theoretical neurobiology from modelling of behaviour using continuous differential Equations (Friston 2005)–like those used in filtering problems in engineering (Kalman and Bucy 1961)–to models predicated upon discrete transitions (Mirza et al. 2018; Schwartenbeck and Friston 2016; Schwartenbeck et al. 2019) of the sort found in partially observed Markov decision processes (Åström 1965)–with applications in artificial intelligence research (Kaelbling et al. 1998).

To gain some intuition as to how sequences can emerge from continuous dynamical behaviour, consider a system involving populations of carnivores, herbivores, and plants (Volterra 1928). As the plant population increases, the herbivore population increases in turn. As they eat the plants, this causes a fall in the plant population. However, the herbivores are themselves eaten by the carnivore population, which increases while inducing a corresponding fall in the herbivore population. The drop in herbivore population has two consequences. It deprives the carnivores of food, so causes their population to decline. It also allows the plants to grow back. This simple example illustrates the basic idea behind the dynamics of sequences. It describes a system that can be characterised as a series of peaks in populations of plants, then herbivores, then carnivores, then plants, then herbivores, and so on. This generalised Lotka-Volterra system is an example of a winnerless competition (Afraimovich et al. 2008), as each population competes to increase its number, but ultimately gives way to another population.

From a neurobiological perspective, this emergence of discrete sequences from continuous dynamics may occur as populations of neurons increase and decrease their firing rates in turn. For recent reviews of neuronal sequence generation, see (Pezzulo et al. 2014; Frölich et al. 2021). Conspicuous examples of this phenomenon include the sequential activation of place cells as animals move through a series of spatial locations (Foster and Wilson 2007; O'Keefe and Dostrovsky 1971; Redish 2016), and during subsequent replay of these sequential activations (Foster 2017; Louie and Wilson 2001; Pezzulo et al. 2017). This decomposition of spatial trajectories into a series of locations offers an explanation for the role of brain oscillations, such as the hippocampal theta rhythm that occurs at approximately the same frequency as sequential place cell activation during behaviour (Buzsáki 2002). Similar dynamics have been observed in brain-wide calcium imaging of Caenorhabditis elegans (Kato et al. 2015).

**Fig. 1:**
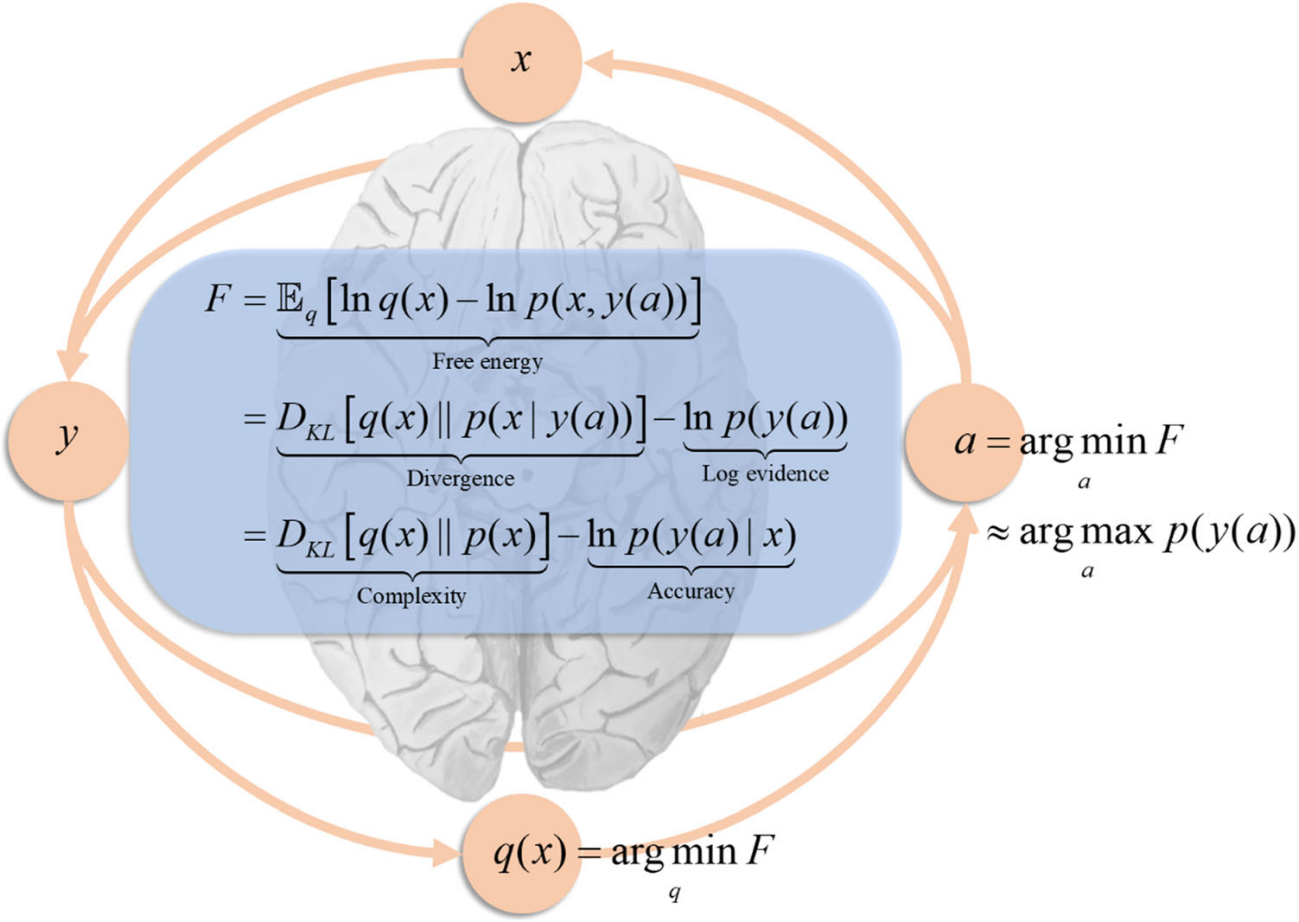
Active inference. This figure summarises the central idea in active inferential formulations of brain function. The pink circles connected by arrows illustrate an action-perception cycle (Fuster 2004) in which states of the external world (*x*) cause sensory data (*y*). Crucially, in active inference, both action and perception minimise a quantity called free energy (*F*), which is shown in the blue panel. Perception corresponds to belief updating and the optimisation of a probability distribution (*q*(*x*)), so that it approximates the posterior probability *p*(*x*|*y*) of states of the external world given the sensory data sample, under an internal (generative) model *p*(*x*,*y*). Action selection corresponds to generating actions (*a*) to change the external world and our sensory input to better conform to our model of the world. See the main text for explanation

In this paper, we discuss the emergence of discrete sequences from continuous neuronal dynamics through the lens of active inference (Friston et al. 2012)—a theoretical framing of behaviour that generalises the Bayesian brain hypothesis (Doya 2007; Knill and Pouget 2004) (see Fig. 1). We provide examples of this process at work in several cognitive domains, including motor control, perception, planning and linguistic communication. The key idea underlying active inference is that our brains behave as if they were using statistical models to explain how sensory data are generated. Among those factors required to explain sensations are the choices we make about where to deploy our sensory epithelia. This means the models our brains use must include the choices and plans we make, when engaging with the world around us. In other words, we model our own agency. Once framed in this way, decisions about what to do next become inference problems (Botvinick and Toussaint 2012). By combining our prior beliefs about how we are likely to respond to the sensations generated by our actions, we arrive at posterior beliefs about the most plausible sequence (plan or policy) we might pursue.

A common alternative to generative modelling approaches is the use of function approximators and neural networks, as in deep learning; and there are several successful examples of sequence learning and prediction for machine learning and data analysis (Sutskever et al. 2014; LeCun et al. 2015). The main advantage to adopting an explicit generative modelling approach is that it is transparent, in the sense that the ensuing computations have a clear interpretation. Realizing interpretable and “explainable” models is essential for the agenda of trying to understand how our nervous systems behave; and also recognized as an important objective to realize future technologies that humans can trust (Castelvecchi 2016; Parr and Pezzulo 2021).

We start with a brief overview of generative models, with a focus on the representation of trajectories with a sequential or ordinal aspect (Dehaene et al. 2015; Friston and Buzsaki 2016). While sequences can be formulated in terms of differential equations in continuous time, their behaviour is often captured more simply through a series of discrete transitions. This is followed by examples in which different sorts of behaviour emerge when these generative models are integrated over time to simulate sentient behaviour. We touch upon formulations of moving and planning of the sort required to generate handwriting and limb movements, and upon forms of communication that range from birdsong to language. As we will see through these examples, one of the key benefits of formulating our internal models in terms of discrete sequences is that it simplifies the process of planning—i.e., the adjudication between alternative future sequences of states.

### Generative models

A generative model specifies the mechanisms by which sensory data are generated. It normally takes the form of a joint probability distribution over the data and the things necessary to explain those data. Figure 1 highlights the central role of a generative model in active inference. In brief, different kinds of creature—or different kinds of person—might expect to encounter different distributions of sensory input, depending upon their preferred environments and behaviour. These distributions can be highly context sensitive and depend upon things that are not directly observed. Such dependencies are captured in the generative model that the creature (or person) uses to explain their sensations. Crucially, it is the creature’s generative model that guides its active engagement with the external environment, i.e., its action-perception cycle (Fuster 2004).

The pink circles connected by arrows in Fig. 1 provide a formal illustration of the action-perception cycle of a generic active inference creature, in which states of the external world (*x*) cause sensory data (*y*). Sensory data could mean patterns of photoreceptor activation, somatosensation, or any other modality. These data induce perceptual belief updating, operationalized as the optimisation of a probability distribution (*q*(*x*)) so that it approximates the posterior probability *p*(*x*|*y*) of states of the external world given the sensory data sample, under an internal (generative) model *p*(*x*,*y*). Given sensory data and our beliefs about their causes, we generate actions (*a*) to change the external world and our sensory input to better conform to our model of the world. Both action and perception minimise a quantity called free energy (*F*) which depends upon our beliefs about the world, our sensory input (and via this on action), and on our internal model. The blue panel unpacks free energy to aid intuition. It can be formulated as the difference between a Kullback–Leibler divergence—which measures the discrepancy between beliefs (*q*(*x*)) and the posterior probability (*p*(*x*|*y*))—and the log evidence or marginal likelihood for a model (*p*(*y*)). When perceptual inference has closed the gap—quantified by the divergence—action maximises the evidence term (i.e., self-evidencing dynamics (Hohwy 2016)). An alternative interpretation of the evidence is as a distribution of sensory data compatible with life, at which point self-evidencing is simply homeostatic control (Cannon 1929). Free energy can also be expressed—from the perspective of Bayesian statistics—as the difference between the complexity of a model and the accuracy with which it predicts data (Penny 2012). In machine learning, it is often referred to as an evidence bound (Winn and Bishop 2005). This emphasises its interpretation as a measure of model fit. The symbol *D*_*KL*_ indicates a Kullback–Leibler divergence. The $\mathbbm{E}$ symbol indicates an expectation (or average).

The perception-as-inference perspective offered by active inference has roots in Helmholtzian psychology and the notion of unconscious inference (Helmholtz 1866) and perception as hypothesis testing (Gregory 1980). In addition to their role in helping us explain our world, generative models specify how we expect our world to be. We can correct deviations from this expectation through acting to change our sensory input, so that it coheres with our model: i.e., the generative model is not just ‘descriptive’ but also ‘prescriptive’, as it guides our behaviour. This is the idea underneath the principle of homeostasis (Cannon 1929), and modern formulations in terms of the underlying stochastic physics (Friston 2019), which says that deviations from some allowable distribution of sensations should be corrected through (active) negative feedback mechanisms. By engaging negative feedback mechanisms—to render sensory data more probable under a generative model—the resulting data then afford better evidence for that model. For this reason, this is sometimes referred to as self-evidencing (Hohwy 2016).

In neurobiology, the above formulation reduces to an account of spinal (e.g., motor) and brainstem (e.g., autonomic) reflexes. By comparing top-down predictions carried in descending motor tracts to the data carried by proprioceptive afferents, these circuits modify the firing rates of motoneurons (Shipp et al. 2013; Adams et al. 2013a). The resulting changes in muscle length brings the proprioceptive signal into line with the top-down predictions (Feldman and Levin 2009). On this view of behaviour, the key problem the brain must solve is how to get the predictions right. This brings us back to the importance of optimising the generative model used to generate these predictions. An important feature of biologically plausible generative models is that they are dynamic. They must account for dynamic changes in the outside world that cause sensory input. In addition, they must account for the actions of the creature employing that generative model. This raises an important question about the ways in which the brain might represent dynamical systems.

**Fig. 2:**
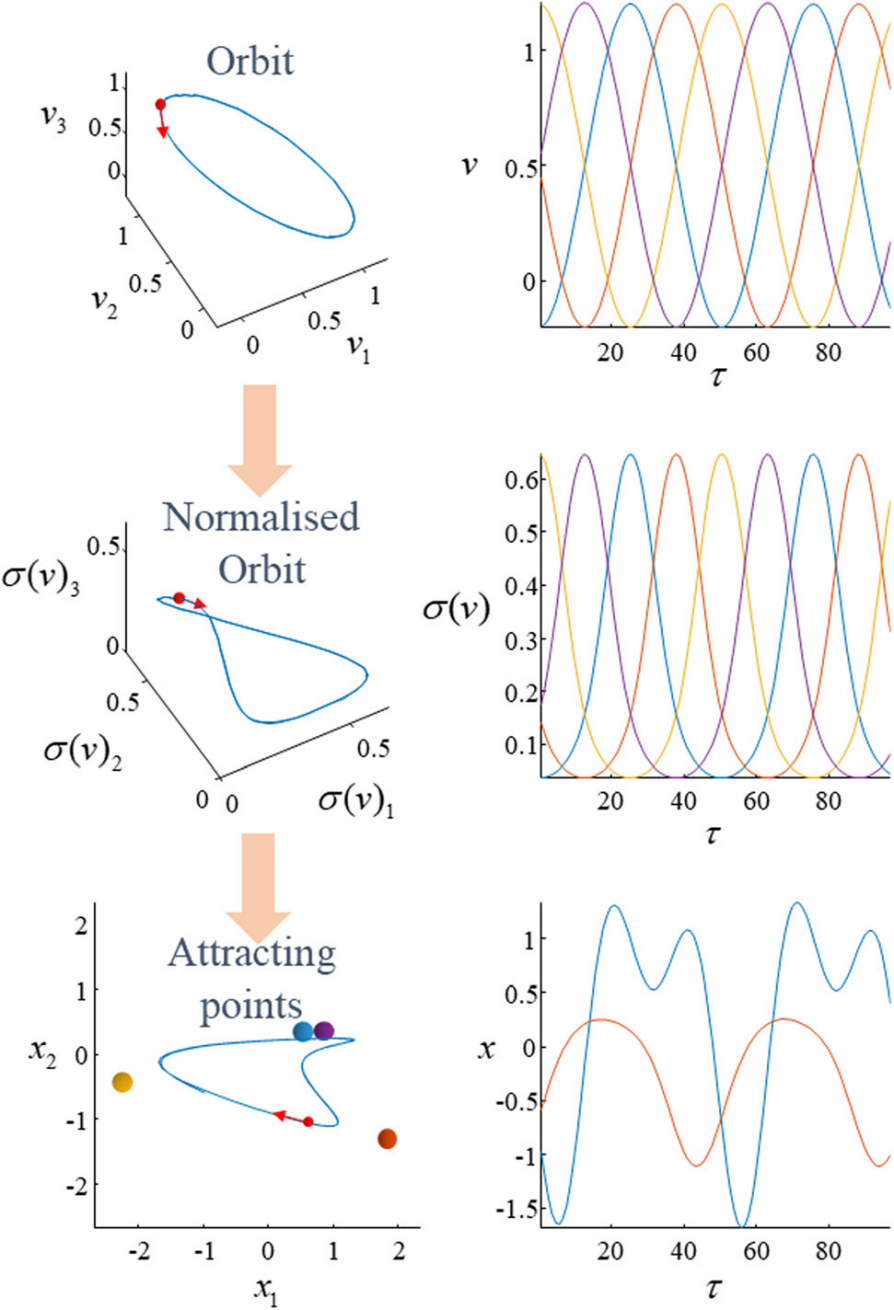
Sequences in continuous dynamical systems. This figure sets out the key theme of this paper. It illustrates the emergence of discrete sequences from continuous dynamical systems, and the way in which these can be used to direct behaviour. The upper left plot shows an orbit in a 4-dimensional space (omitting one of these dimensions for plotting). The upper right plot shows all four coordinates (*v*) of this orbit as they evolve through time. An orbit of this sort can be regarded as a linearised version of a generalised Lotka-Volterra system, which has a saddle point at the origin such that a decrease along one-dimension (e.g., predator population) results in an increase along another dimension (e.g., prey population). The middle plots show a normalised version of this orbit (using the symbol *σ* to indicate a normalisation function), such that the coordinates at each point sum to one. The lower left plot shows the behaviour of a system in which each coordinate of the normalised orbit is associated with the spring constant in one of a set of springs placed in different locations of a 2-dimensional space—all attached to the same particle in this space. This means each spring is sequentially tightened, giving rise to the trajectory of the lower left plot. The evolution of the coordinates of this trajectory is shown in the lower right plot

Figure 2 illustrates the behaviour of an example system that we can use to unpack the central concepts behind these dynamical representations. The construction of the system is described in the legend and results in an orbit (in 4-dimensions) that visits the maximum for each coordinate in turn—much like the ecological example of predators and prey in the introduction. Each coordinate is associated with a point in a 2-dimensional space. A particle in this 2-dimensional space is drawn to each of these points as if attached to a spring connecting it to that location. The spring constants for each point are scaled by the normalised coordinates in the 4-dimensional system. The result is a relatively complex orbit in the 2-dimensional system, in which the particle is sequentially drawn to the different attracting points. This construction represents a method for generating arbitrary orbits, which might differ in the locations of the attracting points or the order in which they are visited.

**Fig. 3:**
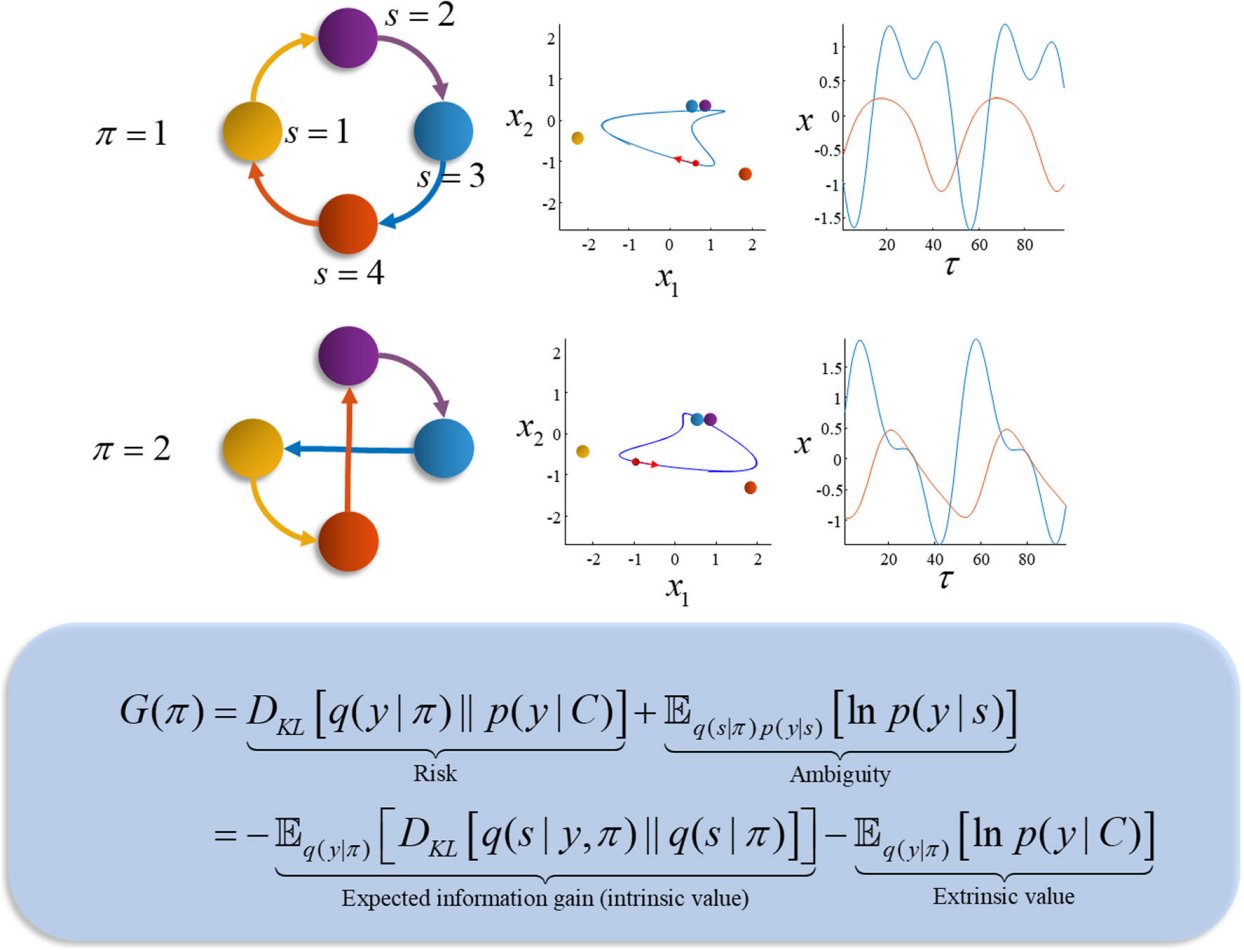
Alternative sequences. This figure illustrates a key problem associated with intelligent behaviour: given multiple possible plans or sequences (e.g., sequences of locations to be visited to reach a destination), how do we select among them? Two possible sequences of transitions between attracting points are given for the system in Fig. 2 through providing two different orientations for the orbit generating the sequence—shown here schematically on the left through the different patterns of transitions between the attracting points (indexed by *s*). These lead to different behaviours for the dynamical system, shown on the right. Interpreting each set of transitions as an alternative behavioural policy (indexed by *π*), we are left with the question as to how to select between policies. Under active inference, this is formulated as a Bayesian inference problem, in which prior beliefs about each policy are scored by the expected free energy (*G*) under that policy. This quantity is shown in the blue panel and is decomposed into two complementary interpretations that are unpacked in the main text. As in Fig. 1, the distributions indicated by *p* represent the generative model, while the distributions indicated by *q* are the beliefs obtained through minimising free energy. The *C* variable is a parameter of a distribution over prior preferences

The system in Fig. 2 is straightforward to represent using continuous differential equations describing the evolution of each part. However, the sequential aspect of these dynamics suggests an alternative, simpler representation. Specifically, we could follow the approach taken in the field of symbolic dynamics (Morse and Hedlund 1938); a branch of dynamical systems theory that assigns symbols to parts of a dynamical state space and represents a trajectory in terms of the transitions of the system from one symbol to another. Figure 3 illustrates this graphically, displaying the orbit in terms of a set of arrows mapping between alternative attracting points. In addition, it shows what happens when we rotate the original 4-dimensional orbit, such that the order in which the attracting points are visited (i.e., the order in which the springs are tightened) changes. The result is a simple representation of the system’s dynamics in terms of a set of allowable transitions.

While complex and interesting dynamics can be generated from sequences of fixed points, these are more akin to the dynamics associated with central pattern generators (Marder and Bucher 2001) than to autonomous, purposeful, behaviour. To make the leap from the former to the latter, we need some way of adjudicating between alternative sequences—like those shown in Fig. 3—given some motivational goal. Under active inference, the drives that motivate choices between alternative sequences are formulated as prior beliefs. This means that motivational drives become part of the generative model, representing explanations^1^ for different behaviours or policies. Just as in Fig. 1 we scored inferences according to their free energy, Fig. 3 shows how we can score policies according to their expected free energy (Friston et al. 2020b). This quantity represents the cost of pursuing a policy, which can be decomposed into a risk and an ambiguity penalty. Formally speaking, the risk is analogous to the complexity cost in Fig. 1. Similarly, ambiguity is (loosely speaking) the expected inaccuracy, where both risk and ambiguity are evaluated under the sensory outcomes that are expected, under the policy in question.

To understand these quantities, we must draw a distinction between those things that can be observed directly (i.e., sensory data) and the hidden states (i.e., explanatory variables) that generate them and must be inferred. Our discussion so far has focused upon the dynamics of hidden states of the world. However, to engage with these states, they must have sensory consequences. Risk is defined as the divergence between a distribution of preferred sensory inputs (e.g., homeostatic set points) and the distribution anticipated under a given policy. Framing this in terms of Fig. 3, if we knew that the yellow state was associated with a distribution of sensory outcomes incompatible with our preferences (i.e., it is risky), we might favour sequences in which the yellow state is omitted or features less frequently. Ambiguity is a measure of the confidence with which we can predict sensory data given the hidden states that caused them. Ambiguity aversion would lead us to favour sequences in which states that precisely predict sensory data feature more frequently than those with a broader distribution of outcomes (Parr and Friston 2017).

An alternative way of carving up the expected free energy is shown in Fig. 3, highlighting its decomposition into intrinsic and extrinsic value terms. Intrinsic value is the information gain expected on pursuing a policy (Lindley 1956). In other words, it is the change in beliefs about hidden states anticipated on observing sensory data, averaged under the distribution of sensory data anticipated under that policy. Maximising this quantity results in explorative behaviour and active sensing (Mirza et al. 2016, 2018; Itti and Baldi 2006; Itti and Koch 2000; Yang et al. 2016; Donnarumma et al. 2017). Extrinsic value represents degree to which anticipated data cohere with the creature’s preferences. This quantity promotes exploitative behaviour (Todorov 2007; Kinjo et al. 2013) of the sort that predominates in most reinforcement learning settings (Sutton and Barto 1998). In sum, expected free energy balances exploratory and exploitative drives through combining them into a single quantity. When there is a stark difference between preferences for outcomes under different policies, the exploitative part dominates. In uncertain contexts, the explorative part supervenes. Taken together, these contributions to the expected free energy introduce agency into the model, expressing a prior belief that we will act in a goal-directed and information seeking, curiosity-driven manner.

**Fig. 4:**
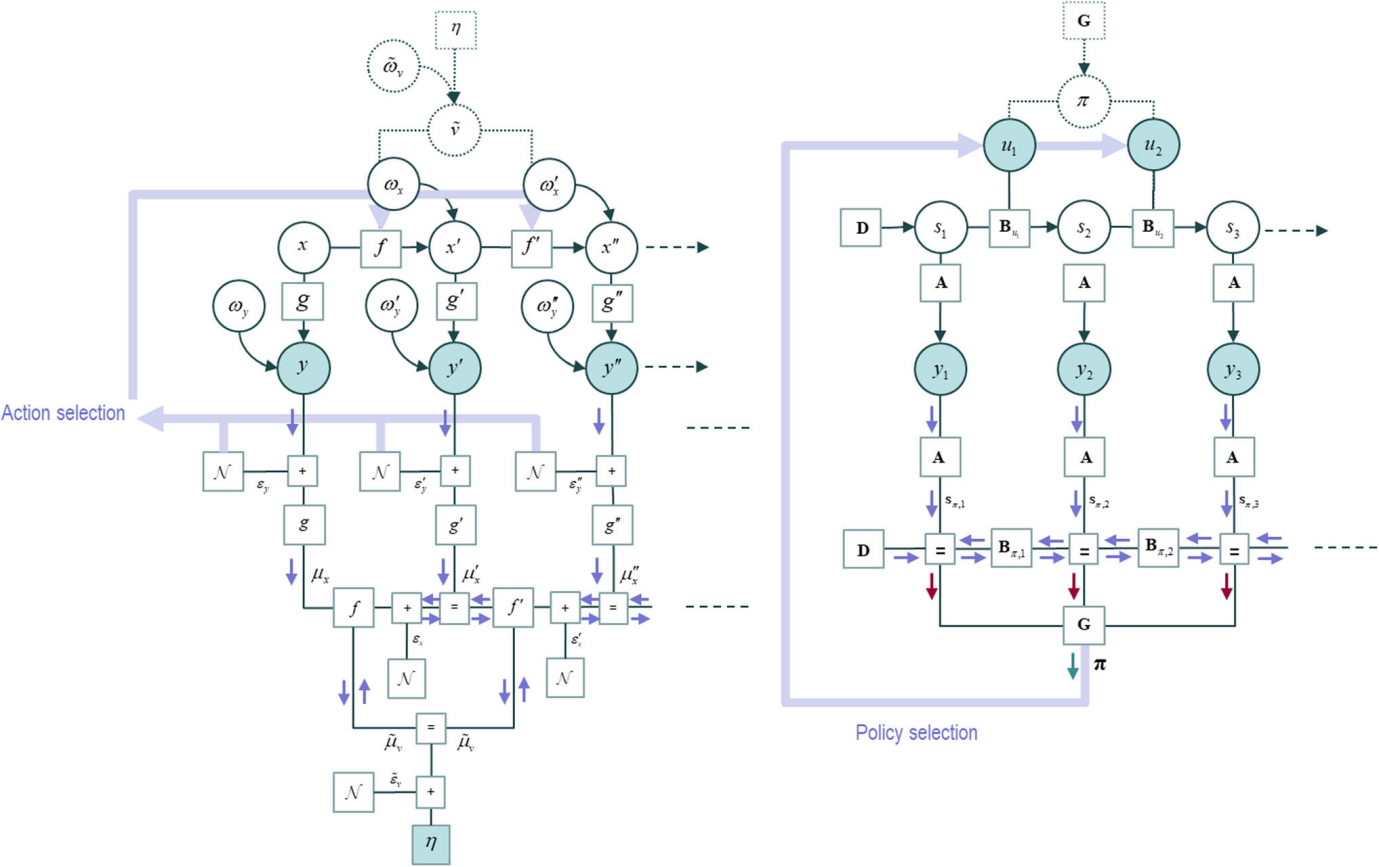
Generative models for continuous and discrete time. This figure, adapted from Friston et al. (2017b) to which we refer readers for technical details, illustrates two ways of formulating a generative model of a dynamic process. Everything above the sensory data nodes (*y*) is the generative model. Everything below represents the inversion of the generative model. The models are shown as factor graphs (Loeliger et al. 2007). These use squares to indicate factors of a probability distribution, and circles to indicate the variables connected by those factors. For instance, the square labelled *g* in the left plot indicates the probability of data given continuous states (*x*). Similarly, those labelled **A** in the model on the right indicate the probability of data conditioned upon discrete states (*s*). The prime (′) notation in the model on the left indicates a temporal derivative. This means the factors connecting states in the horizontal direction (*f*, *f*′, *f*′′, …) represent the relationship between position, velocity, acceleration, and so on—effectively providing a summary of a trajectory based upon the coefficients of a Taylor series expansion. In contrast, the subscripts in the model on the right relate to the discrete time step associated with that state. As such, the transition probabilities (**B**) tell us the probability that a state takes on a particular value one step in the future, given the present state. This transition is itself conditional upon the action (*u*) selected at that time that, in turn, is determined by the policy (*π*). The *ω* symbols are normally distributed fluctuations. The *E* symbol indicates a zero-mean normal distribution. The blue arrows indicate the message passing required to minimise free energy. The red arrows show those messages needed to compute the expected free energy and, via this, the expected policy (**π**). The form of these messages take include prediction errors (*ε*) and expectations (*µ* or **s**) and underwrite theories of neurobiological message passing schemes such as predictive coding (Friston and Kiebel 2009; Rao and Ballard 1999; Srinivasan et al. 1982) and those based on variational message passing (Parr et al. 2019; Friston et al. 2017c; Dauwels 2007)

Figure 4 summarises the anatomy of two sorts of generative model based upon alternative representations of dynamical systems, formulated in continuous time (left) and discrete time (right). The details are unpacked in the legend, but the key points are as follows. Each model provides a graphical expression for the factors of the joint probability distribution over hidden states and sensory data. In Fig. 4, everything above the sensory data nodes (y) is the generative model. Everything below illustrates the structure of the inversion scheme used to solve the inference problem is shown. This mirrors the structure of the generative model and depends upon the passing of messages (shown as blue arrows) among nodes of a network. Message passing of this sort has been associated with the dynamics of neuronal networks, where messages are passed across synapses (Friston et al. 2017a; Parr et al. 2019; Parr et al. 2022; Pezzulo et al. 2018). The generative model shown on the left of Fig. 4 is formulated in continuous time, using the coefficients of a Taylor series expansion of the coordinates of some system to represent a trajectory (Friston et al. 2010). The model on the right is formulated as a sequence of transitions, with discrete time steps. The transition probabilities vary with the policy selected, where the prior over policies is determined by their negative expected free energy.

Each of these models can be extended to an arbitrary level of complexity, through addition of additional dimensions or factors of hidden state variables, or through hierarchical composition of the models of Fig. 4 to account for separable timescales of the modelled dynamics. For details on hierarchical expansion of these models, please see (Friston et al. 2017b). Having said this, it is normally desirable to limit model complexity unless an increase in complexity affords a corresponding increase in the accuracy with which data can be explained (Jefferys and Berger 1992). In subsequent sections, we draw from examples in computational neuroscience in which these two (continuous and discrete) approaches have been adopted.

### Moving and planning

This section moves from the relatively abstract treatment above and showcases two examples in which generative models have been used as a basis for autonomous motor behaviour and planning. First, we describe a generative model that has been used to simulate the complex trajectories required for handwriting (Friston et al. 2011). This is based upon the kinds of sequences we saw in Fig. 2. We then turn to a more deliberative example in which the sequence-generating part of the dynamics were replaced with a discrete-time model to simulate a simple coordination task (Parr et al. 2021). Together, these demonstrate the autonomous behaviour that can be developed from models comprising sequences of attracting points, and the way in which we can efficiently select between alternative sequences once formulated in discrete time intervals. Furthermore, these two examples illustrate that active inference models can be used to simulate the neuronal dynamics that one should expect to observe under the hypothesis that the brain maintains a generative model of sequential dynamics. These include the winnerless competition between neural populations that we might anticipate given sequences of attracting points, and the periodic belief updating—manifesting as local field potentials—we would expect given a model articulated in terms of a discrete sequences.

**Fig. 5:**
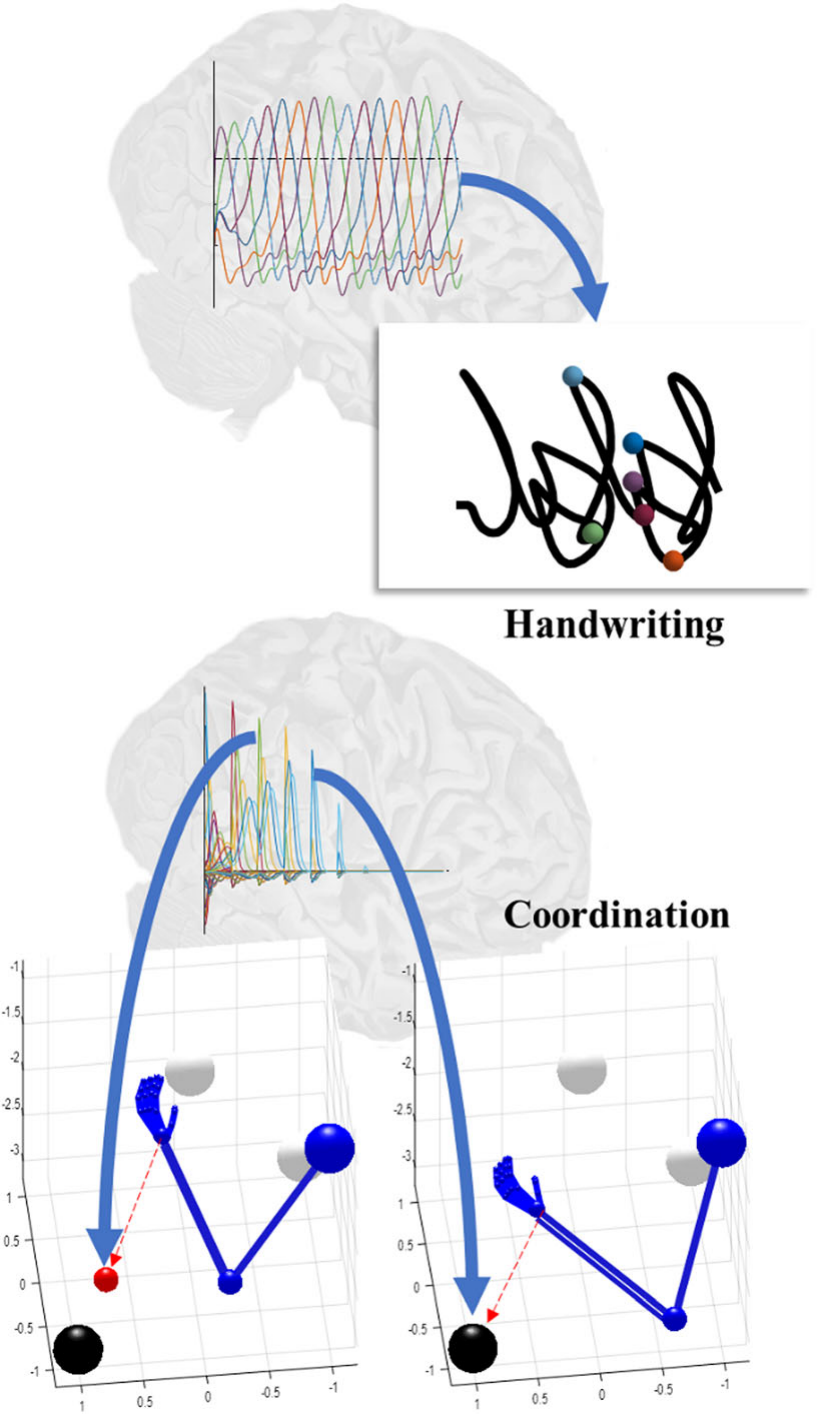
Sequential movements. This figure illustrates the use of sequences of fixed points to develop complex motor behaviours. The upper part of this figure illustrates handwriting behaviour, resulting from inversion of a generative model. The sequential Lotka–Volterra dynamics are shown superimposed on the image of the brain, with each peak mapped to the attracting point with the corresponding colour in the sample of synthetic handwriting shown. The handwriting is generated by visiting the series of attracting points, assuming the paper beneath the hand is moved to the left as the arm continues to write. The lower plot shows motor control using an arm with three degrees of freedom. The plot superimposed upon the brain shows simulated local field potentials, generated as described in Friston et al. (2017c). They represent the updating of beliefs about the fixed points selected at each discrete time point. The frames shown below illustrate the fictive fixed points selected by the discrete model, and the movement of the arm towards these points. The graphics for these figures are adapted from the simulations described in Friston et al. (2011) (handwriting) and in Parr et al. (2021) (coordination). The winnerless competition superimposed on the upper image and the local field potentials superimposed on the lower image represent the different kinds of neural responses we might expect to measure under each sort of generative model

The handwriting model is based upon the generative model and accompanying message passing (i.e., belief updating) illustrated on the left of Fig. 4. The first thing we need to do—to understand this model—is to consider the sorts of sensory data we would have to explain on observing ourselves writing. This includes both proprioceptive signals as we move our joints, and visual input, as we see our arm and hand moving. So, what are the explanatory variables we need to account for these sensations? They are simply the angles of the joints in our arm and their instantaneous rates of change—i.e., the hidden states. However, a static mapping from these variables to our visual and proprioceptive sensations is not enough to explain the trajectories we would expect on tracing out letters. For this, we need to incorporate dynamics in these hidden states. This involves equipping the model with a set of abstract hidden states that evolve according to the Lotka-Volterra equations. These are developed from the same sort of predator–prey interactions described in the introduction. An example of the inferred trajectory of such states is more superimposed on the image of the brain in the upper part of Fig. 5. Just as in Fig. 2, the sequential peaks in these states can be associated with fictive attractors to which the arm is pulled. These are fictive in the sense that there are no springs in the real world pulling our arm as we write. However, if incorporated in the generative model, we can predict the dynamics that would result if such attractors were pulling our hand around the letters. Finally, low level reflexes cause the data that would be explained by this model. In other words, the arm moves to fulfil proprioceptive and visual predictions—which represent the anticipated data on engaging in writing behaviour. An example trajectory is shown in the upper part of Fig. 5.

Implicit in this perspective on handwriting, and other complex motor trajectories, is the idea that movement can be chunked into a sequence of short trajectories (Wymbs et al. 2012) and micromovements (Navas and Stark 1968; Miall et al. 1993; Woodworth 1899; Doeringer and Hogan 1998). Given this, we could simply omit the additional hidden state dimensions required to generate the sequential dynamics, and work in discrete time instead as in the right of Fig. 4. On doing so, it becomes simpler to adjudicate between alternative transition probabilities, and to generate more purposeful behaviour. As an example, consider the problem shown in the lower part of Fig. 5. Here we have an arm in a 3-dimensional space, and three possible targets depicted as black and white spheres. One of the three spheres is black at any given time, but this can swap between spheres periodically. The task is to move the hand to the black sphere. How do we formulate this task as a generative model? As in the handwriting example, we must first identify the data we must explain. In addition to the proprioceptive and visual data pertaining to the arm, we also need to explain the visual data from the target spheres.

The model developed to solve this task combined a continuous model, like that described for the handwriting model, with a discrete time model. This has the effect of piecing together a sequence of continuous trajectories. At each discrete time step, a new attracting point and target (black) sphere was predicted. However, there was no pre-defined sequence of attracting points as in the handwriting example. More precisely, there were several possible sequences of points (i.e., policies) that could have been chosen. To adjudicate between these, the expected free energy of each sequence was computed, under the prior belief (or preference) that the hand location is the same as that of the target sphere. This meant those policies involving sequences of points—that ended at the target sphere—had a lower expected free energy, rendering them more plausible. Figure 5 illustrates two frames in which a fictive attracting point is first placed at an intermediate point between the hand and the sphere, and then at a point coinciding with the sphere as the hand is drawn towards it. As above, the predicted attracting points have consequences for proprioceptive predictions, which themselves are fulfilled through spinal reflex arcs.

There are many applications of generative models in motor control. Other examples we could have chosen include the use of Lotka-Volterra systems to time responses in a cerebellar conditioning paradigm (Friston and Herreros 2016), the use of harmonic oscillators to model whisker dynamics (Mannella et al. 2021), or the use of attracting points in motivating reaching behaviours in a robotic system (Pio-Lopez et al. 2016). We have chosen two examples that emphasise sequential processing, and the emergence of purposeful autonomous behaviour. The handwriting example illustrated how visiting a series of attracting points in sequence lets us specify arbitrary trajectories. The coordination task goes beyond this to the adjudication between alternative sequences to fulfil a goal—i.e., planning.

### Language and communication

In this section, we turn to the application of active inference to communication, to show that the same computational principles that we have used to explain perceptual and motor control might also underlie advanced forms of (social) cognition. We highlight the recent move from accounts of communication in terms of coupled dynamical systems—of the sort used to simulate birdsong—to more linguistic communication defined in terms of sequences of words intended to convey meaning. First, we must specify what is meant by communication. There must be at least two creatures, such that each has a conspecific to communicate with. Each must be able to take action that influences the sensorium of the other. Successful communication results in a (generalised) synchronisation of beliefs between the two–or in the formation of a common ground in psycholinguistics parlance (Clark and Brennan 1991).

**Fig. 6:**
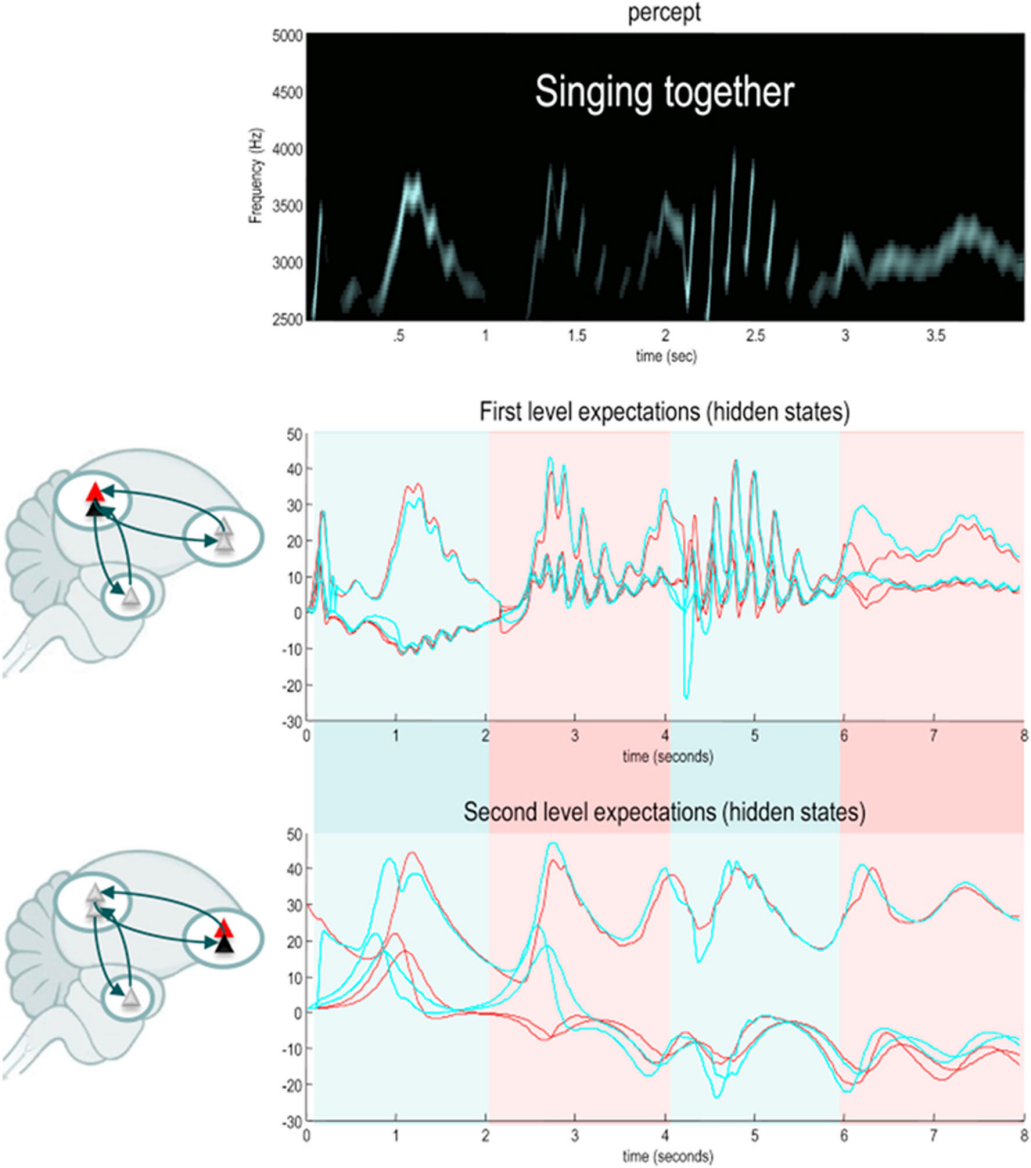
Synthetic birdsong. This figure, reproduced from Friston and Frith (2015), illustrates a simple form of communication based on a generative model for birdsong. The underlying generative model, shared between two birds, uses two chaotic (Lorenz) dynamical systems. At the second level, a slow Lorenz attractor predicts parameters of the faster Lorenz attractor at the first level, which itself predicts sonographic data. By minimising free energy through action, the birds reflexively generate the songs they expect to hear. This means generating no sound when one bird hears the other sing and singing when there is silence. Minimising free energy through perceptual inference allows the birds to synchronize their internal narrative, such that the segments of the song they sing in turn cohere as part of a single (musical) narrative. The blue and red lines in the middle and lower plots indicate the beliefs (expectations) held by each of the two birds about the states of the Lorenz systems. The shading indicates which of the two is singing at each point. Note the synchronisation between the beliefs of the two birds based upon the auditory data they take turns to generate

Figure 6 illustrates the behaviour of a pair of songbirds. Their generative models—which are identical to one another—are based upon coupled Lorenz attractors (Kiebel et al. 2009). Lorenz systems exhibit a chaotic itinerancy that lends them an appearance of autonomy (Kuznetsov et al. 2020; Lorenz 1963). The generative model used by the songbirds to explain auditory and proprioceptive (laryngeal) data comprises a high-level Lorenz system (second level hidden states) that predicts the parameters of a low-level Lorenz system (first level hidden states), which causes auditory data. When one bird hears the other singing, it updates its beliefs about the hidden states causing these data. When it hears nothing, brainstem reflexes cause the bird to produce the sounds predicted by its model. In Fig. 6, the birds take turns to sing. The key insight from this figure is that the auditory data produced by the two birds is sufficient for the two to synchronise their expectations about the states of the generative model. Put simply, both birds are trying to predict their auditory sensations and both birds (take it in turn to) generate those sensations. Maximum predictability is assured when they both predict and generate the same thing.

**Fig. 7:**
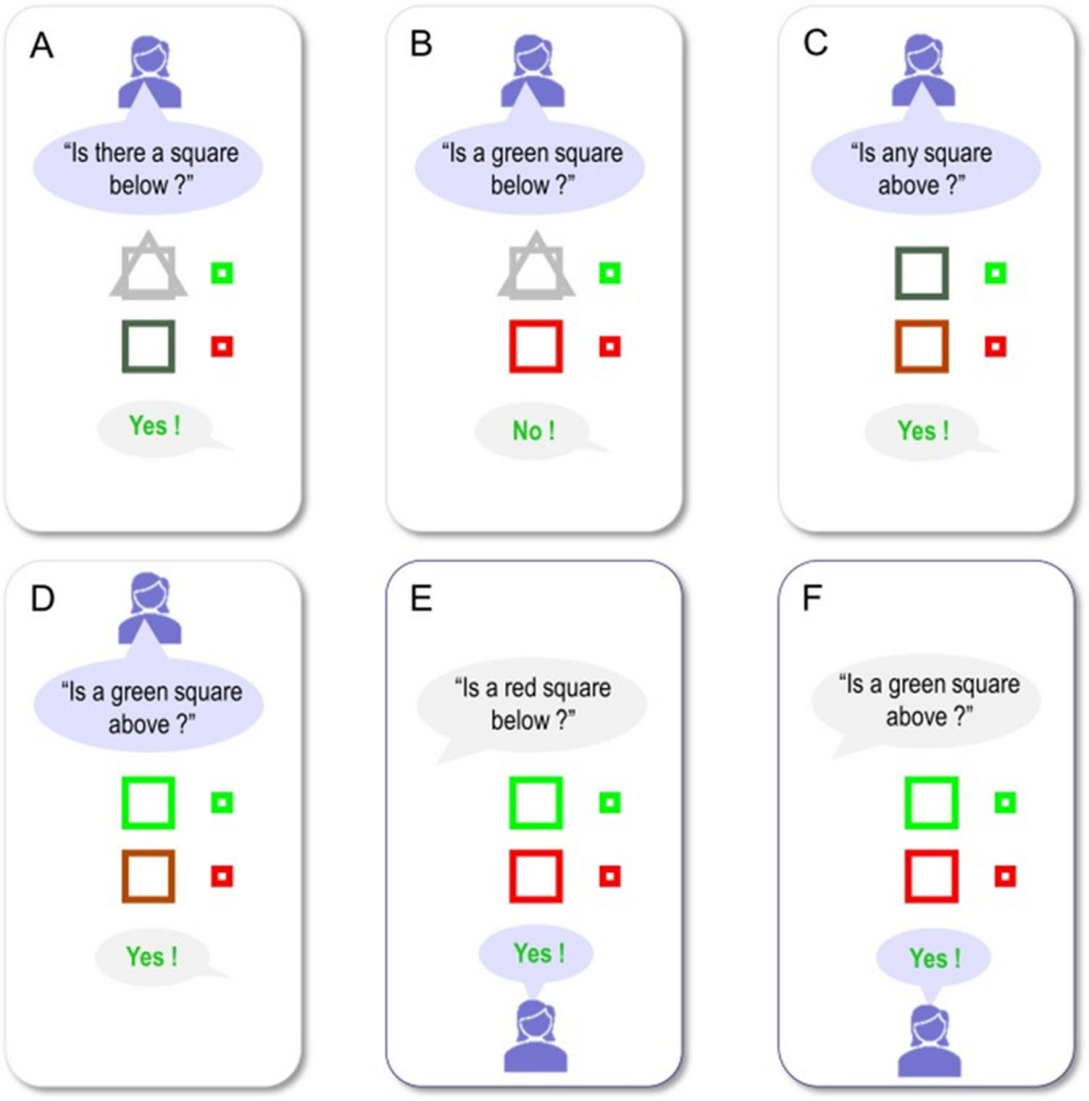
Linguistic communication. This figure, reproduced from Friston et al. (2020a), illustrates a simulation of a simple game based upon 20 questions. The agent is represented by the silhouette icon, and her beliefs are illustrated by the central scene in each panel with the two shapes. The correct scene is indicated by the green and red squares on the right of each panel. As she asks a series of questions (panels A-D), her beliefs about the scene resolve into veridical beliefs, at which point she can correctly answer two questions about the scene (panels **E**–**F**)

This communication by synchronisation again depends upon the continuous time generative model shown on the left of Fig. 4. However, there is much to be gained through moving to discrete time models in understanding linguistic communication. Partly, this is for the same reason as we saw in the context of movement: it is simpler and more efficient to select among alternative sequences than trajectories that vary along continuous dimensions. However, it is also due to the discrete nature of language, which comprises units such as phonemes, words, sentences, and so on (Chomsky 2017). Figure 7 reproduces the results of a simulation from Friston et al. (Friston et al. 2020a) that exploits this discrete form. The set-up involves a pair of agents who can ask and respond to questions to find out about a spatial scene, in the style of a game of 20 questions. The underlying generative model is a discrete time model of the form shown on the right of Fig. 4. The hidden states are organised into those that evolve over a slow and a fast timescale. The slower timescale includes the narrative structure (i.e., whether the question currently being asked or answered), the topic of the question being asked (e.g., about shapes, locations of objects in a scene, or their colour), the structure of the scene, and the relevant nouns, adjectives, and prepositions that can be slotted into the question. These combine to generate sequences of hidden states at the faster timescale, with an appropriate syntactic structure and semantic content. From these, the spoken words can be predicted.

By selecting between alternative sequences for the topic of the question and the specific words to slot into this, the agents can solicit answers from one another. In the motor control example above, the selection between these sequences was driven mostly by the preference for reaching the target. This depended upon the extrinsic value or risk term in the expected free energy. However, in this linguistic setting, questions are driven purely to gain information—emphasising the intrinsic or epistemic value in the expected free energy. Figure 7 shows how an agent, initially uncertain about the scene, asks a series of questions that resolve her uncertainty about the objects in the scene such that, when queried, she can accurately answer questions about the scene.

As in the previous section, these two examples—the songbirds and the question game—were chosen to illustrate the expression of autonomous dynamics in a continuous generative model, and the explicit discretisation into sequences that have been employed in more recent models. Both forms of models have found broad application, including in computational psychiatry. For example, the songbird model has been developed to explain pathologies of inference, such as auditory hallucinations of the sort found in schizophrenia (Adams et al. 2013). Discrete models based upon linguistic communication have furthered this, focusing upon the verbal (Benrimoh et al. 2018) and context sensitive (Benrimoh et al. 2019) aspects of these hallucinations in psychosis.

To this point, we have ignored a (possibly ubiquitous) issue surrounding the use of temporally discrete generative models to account for sequential dynamics. This is that each element of the sequence, once expressed in the continuous domain, may have a different temporal duration. Language provides an intuitive example of this problem: different words take different lengths of time to vocalise. Inferences about sequential causes of continuous data therefore require segmentation of that sequence. This problem has been addressed under the same (active inference) framework detailed above in the context of active listening (Friston et al. 2021). The idea is that, by comparing the marginal likelihood—or free energy approximations to this quantity—of alternative boundaries for words in a continuous auditory stream, we can select the boundary associated with the greatest marginal likelihood. Effectively, this treats alternative word boundaries as hypotheses. Comparison of the evidence for each hypothesis allows us to select the most parsimonious segmentation.

**Fig. 8:**
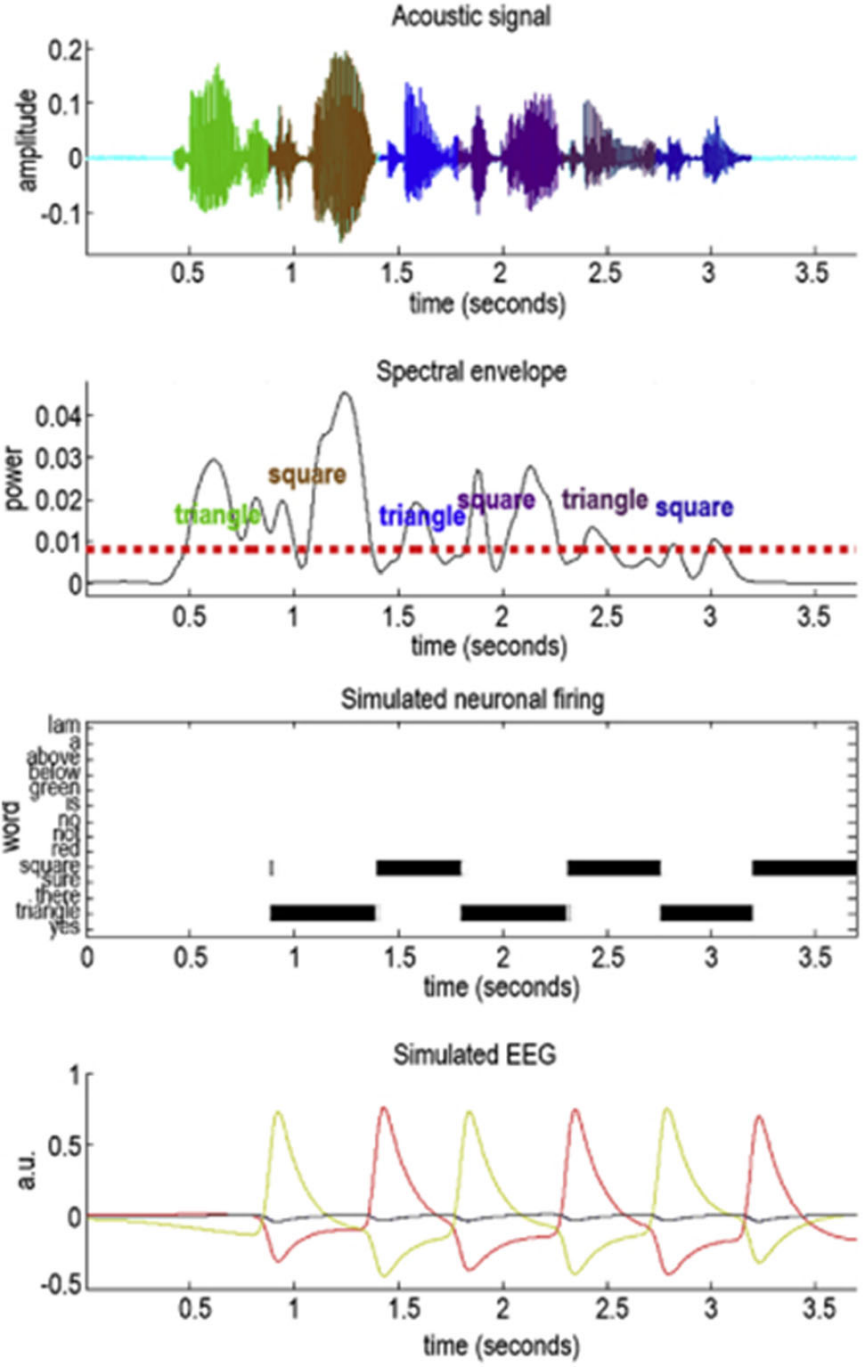
Active listening. These plots illustrate the inversion of a generative model designed to explain auditory data, and is adapted from Friston et al. (2021). The generative model includes a small repertoire of words, shown in the rows of the third plot, each of which predicts an acoustic signal. The first plot shows an acoustic timeseries in which a speaker alternates between the words ‘square’ and ‘triangle.’ The second plot shows the spectral envelope and the threshold (red dotted line) used to identify plausible segmentation boundaries. The third plot illustrates a series of simulated neural populations (rows), each representing a word. Given a prior belief that shape-related words are more plausible, these neurons fire in proportion to the posterior probability of each word at each time. Note the duration of the inferred words differs, but that they are non-overlapping (i.e., the probability sums to one) indicating the successful discretisation of the continuous sequence. The simulated electroencephalographic (EEG) signal in the lower plot is equivalent to the simulated local field potentials in Fig. 5 and represents the rate at which beliefs about each word are updated

Figure 8 illustrates this for an acoustic signal in which a speaker repeats the words ‘triangle’ and ‘square’ and the listener attempts to identify the words out of a (small) vocabulary. Plausible segmentation boundaries are identified based upon a thresholding of the spectral envelope, and then are compared to arrive at a segmentation. The simulated neuronal firing plot illustrates the inferential solution to this problem, with neural populations representing alternative words. These represent each word for different lengths of time but are non-overlapping—illustrating the segmentation of continuous data into a discrete sequence. While applied in the context of listening, the principles underneath this segmentation are generic, reflecting the fact that the problem of segmenting continuous streams of observations into discrete events is ubiquitous in cognitive processing (Kurby and Zacks 2008). These Bayesian principles depend upon the idea that the brain employs a generative model to explain continuous data whose causes have a sequential aspect, and that the model can be optimised by maximising its marginal likelihood.

## Conclusion

This paper set out to showcase the use of generative models that, under active inference, give rise to autonomous behaviour. In brief, this depends upon the idea that creatures implicitly employ models that help them to explain their sensory input and to help guide action. More formally, this means perception and action both contribute to the maximisation of the marginal likelihood of an implicit generative model—via variational approximations to this quantity. Our focus has been on the notion that many cognitive processes depend upon discrete sequences, and that this is reflected in the form of the dynamics in generative models. This includes the predator–prey like dynamics that mediate a winnerless competition in continuous generative models. However, it also includes the more explicit discrete time models that are used to account for planning and decision-making. These alternative framings of the dynamics have consequences for the anatomy of the neuronal circuits solving generative models, and for the activity of the neurons in these circuits. The abstract notion that sequential dynamics are an important part of our internal models gets us only so far. To make this idea useful, it is necessary to commit to specific forms of generative model to explain specific behaviours or neuronal responses. To this end, we outlined examples in motor control and linguistic communication that exploit these sequential dynamics, and briefly touched upon the challenge of segmenting a continuous timeseries into a sequence. These included modelling of the complex trajectories of the sort observed in handwriting using, a 3-dimensional reaching task for a 2-jointed arm, the emergence of generalised synchrony through song, a simple language game, and the segmentation of words from a continuous stream of auditory data. However, the same perspective can be applied to any other kind of sequential dynamics. Much of the complexity consists in specifying or learning an appropriate generative model for the task at hand. When the generative model is in place, the behavioural and neural dynamics of any active inference system are reproduced using exactly the same (variational) inference scheme, which is computationally efficient and can be in principle extended to deal with large state spaces (Maisto et al. 2021). Central to all the above is the idea that cognitive systems are self-evidencing. This does not just mean that we behave in such a way as to maximise evidence for some implicit generative model. It also implies that the model must include ourselves, the decisions we make, and the sequences of events these cause.

## Footnotes

1 It may seem unintuitive to frame motivational drives as explanations for behaviour as opposed to the things guiding selection between alternative behaviours. However, this inversion is central to the active inferential formalism. It speaks to the fact that when a motivational drive causes one to behave in a particular way, that drive becomes an explanation for the behaviour.

## References

[CR1] Adams R, Stephan K, Brown H, Frith C, Friston K (2013) The computational anatomy of psychosis. Front Psychiatry. 10.3389/fpsyt.2013.00047 23750138 10.3389/fpsyt.2013.00047 PMC3667557

[CR2] Adams RA, Shipp S, Friston KJ (2013a) Predictions not commands: active inference in the motor system. Brain Struct Funct 218:611–643. 10.1007/s00429-012-0475-5 23129312 10.1007/s00429-012-0475-5 PMC3637647

[CR3] Afraimovich VS, Rabinovich MI, Varona P (2004) Heteroclinic contours in neural ensembles and the winnerless competition principle. Int J Bifurc Chaos 14:1195–1208. 10.1142/s0218127404009806

[CR4] Afraimovich V, Tristan I, Huerta R & Rabinovich MI (2008) Winnerless competition principle and prediction of the transient dynamics in a Lotka–Volterra model. *Chaos: An Interdiscip J Nonlinear Sci*, **18**, 43103, 10.1063/1.2991108. 10.1063/1.2991108 19123613

[CR5] Åström KJ (1965) Optimal control of Markov processes with incomplete state information. J Math Anal Appl 10:174–205. 10.1016/0022-247X(65)90154-X

[CR6] Benrimoh D, Parr T, Vincent P, Adams RA, Friston K (2018) Active inference and auditory hallucinations. Comput Psychiatry 2:183–204. 10.1162/cpsy_a_00022 10.1162/cpsy_a_00022 PMC6317754 30627670

[CR7] Benrimoh D, Parr T, Adams RA, Friston K (2019) Hallucinations both in and out of context: an active inference account. PLoS ONE 14:e0212379. 10.1371/journal.pone.0212379 31430277 10.1371/journal.pone.0212379 PMC6701798

[CR8] Botvinick M, Toussaint M (2012) Planning as inference. Trends Cogn Sci 16:485–488. 10.1016/j.tics.2012.08.006 22940577 10.1016/j.tics.2012.08.006

[CR9] Buzsáki G (2002) Theta oscillations in the hippocampus. Neuron 33:325–340. 10.1016/S0896-6273(02)00586-X 11832222 10.1016/s0896-6273(02)00586-x

[CR10] Cannon WB (1929) Organization for physiological homeostasis. Physiol Rev 9:399–431. 10.1152/physrev.1929.9.3.399

[CR11] Castelvecchi D (2016) Can we open the black box of AI? Nat News 538:20 10.1038/538020a 27708329

[CR12] Chomsky N (2017) The language capacity: architecture and evolution. Psychon Bull Rev 24:200–203. 10.3758/s13423-016-1078-6 27368638 10.3758/s13423-016-1078-6

[CR13] Clark HH & Brennan SE (1991) in Perspectives on socially shared cognition*.* 127–149 (American Psychological Association, 1991).

[CR14] Dauwels, J. in Information theory, 2007. ISIT 2007. IEEE international symposium on*.* 2546–2550 (IEEE).

[CR15] Dehaene S, Meyniel F, Wacongne C, Wang L, Pallier C (2015) The neural representation of sequences: from transition probabilities to algebraic patterns and linguistic trees. Neuron 88:2–19. 10.1016/j.neuron.2015.09.019 26447569 10.1016/j.neuron.2015.09.019

[CR16] Doeringer JA, Hogan N (1998) Intermittency in preplanned elbow movements persists in the absence of visual feedback. J Neurophysiol 80:1787–1799. 10.1152/jn.1998.80.4.1787 9772239 10.1152/jn.1998.80.4.1787

[CR90] Donnarumma F, Costantini M, Ambrosini E, Friston K, Pezzulo G (2017) Action perception as hypothesis testing. Cortex 89:45–60 10.1016/j.cortex.2017.01.016 PMC5383736 28226255

[CR17] Doya K (2007) Bayesian brain: Probabilistic approaches to neural coding. (MIT press, 2007).

[CR18] Feldman AG & Levin MF (2009) in: Progress in motor control: a multidisciplinary perspective (ed Dagmar Sternad) 699–726 (Springer US, 2009). 19227491

[CR19] Foster DJ (2017) Replay comes of age. Annu Rev Neurosci 40:581–602. 10.1146/annurev-neuro-072116-031538 28772098 10.1146/annurev-neuro-072116-031538

[CR20] Foster DJ, Wilson MA (2007) Hippocampal theta sequences. Hippocampus 17:1093–1099. 10.1002/hipo.20345 17663452 10.1002/hipo.20345

[CR21] Friston K (2005) A theory of cortical responses. Philos Trans R Soc Lond B Biol Sci 360:815–836. 10.1098/rstb.2005.1622 15937014 10.1098/rstb.2005.1622 PMC1569488

[CR22] Friston K, Buzsaki G (2016) The functional anatomy of time: what and when in the brain. Trends Cogn Sci. 10.1016/j.tics.2016.05.001 27261057 10.1016/j.tics.2016.05.001

[CR23] Friston KJ, Frith CD (2015) Active inference, communication and hermeneutics(). Cortex A J Devoted Study Nerv Syst Behav 68:129–143. 10.1016/j.cortex.2015.03.025 10.1016/j.cortex.2015.03.025 PMC4502445 25957007

[CR24] Friston K, Herreros I (2016) Active inference and learning in the cerebellum. Neural Comput 28:1812–1839. 10.1162/NECO_a_00863 27391681 10.1162/NECO_a_00863

[CR25] Friston K, Kiebel S (2009) Predictive coding under the free-energy principle. Philos Transact Royal Soc B Biol Sci 364:1211 10.1098/rstb.2008.0300 PMC2666703 19528002

[CR26] Friston K, Mattout J, Kilner J (2011) Action understanding and active inference. Biol Cybern 104:137–160. 10.1007/s00422-011-0424-z 21327826 10.1007/s00422-011-0424-z PMC3491875

[CR27] Friston K, Samothrakis S, Montague R (2012) Active inference and agency: optimal control without cost functions. Biol Cybern 106:523–541 22864468 10.1007/s00422-012-0512-8

[CR28] Friston KJ, Rosch R, Parr T, Price C, Bowman H (2017a) Deep temporal models and active inference. Neurosci Biobehav Rev 77:388–402. 10.1016/j.neubiorev.2017.04.009 28416414 10.1016/j.neubiorev.2017.04.009 PMC5461873

[CR29] Friston KJ, Parr T, de Vries B (2017b) The graphical brain: belief propagation and active inference. Netw Neurosci 1:381–414. 10.1162/NETN_a_00018 29417960 10.1162/NETN_a_00018 PMC5798592

[CR30] Friston K, FitzGerald T, Rigoli F, Schwartenbeck P, Pezzulo G (2017c) Active inference: a process theory. Neural Comput 29:1–49. 10.1162/NECO_a_00912 27870614 10.1162/NECO_a_00912

[CR31] Friston KJ et al (2020a) Generative models, linguistic communication and active inference. Neurosci Biobehav Rev 118:42–64. 10.1016/j.neubiorev.2020.07.005 32687883 10.1016/j.neubiorev.2020.07.005 PMC7758713

[CR32] Friston KJ et al (2021) Active listening. Hear Res 399:107998. 10.1016/j.heares.2020.107998 32732017 10.1016/j.heares.2020.107998 PMC7812378

[CR33] Friston K (2019) A free energy principle for a particular physics. *arXiv preprint *arXiv:1906.10184.

[CR34] Parr T & Friston, KJ (2017) Uncertainty, epistemics and active inference. J R Soc Interface, **14**. 10.1098/rsif.2017.0376 PMC5721148 29167370

[CR35] Friston K, Stephan K, Li B & Daunizeau J (2010) Generalised filtering. Math Problems Eng, **2010**

[CR36] Friston K, Da Costa L, Hafner D, Hesp C & Parr T (2020b) Sophisticated inference. *arXiv preprint *arXiv:2006.04120 10.1162/neco_a_01351 33626312

[CR37] Frölich S, Marković D, Kiebel SJ (2021) Neuronal sequence models for bayesian online inference. Front Artfi Intell. 10.3389/frai.2021.530937 10.3389/frai.2021.530937 PMC8176225 34095815

[CR38] Fuster JNM (2004) Upper processing stages of the perception–action cycle. Trends Cogn Sci 8:143–145. 10.1016/j.tics.2004.02.004 15551481 10.1016/j.tics.2004.02.004

[CR39] Gregory RL (1980) Perceptions as hypotheses. Phil Trans r Soc Lond B 290:181–197. 10.1098/rstb.1980.0090 6106237 10.1098/rstb.1980.0090

[CR40] Helmholtz H v (1866) In: Treatise on physiological optics Vol. 3 (ed J. P. C. Southall) (Dover, 1866).

[CR41] Hohwy J (2016) The self-evidencing brain. Noûs 50:259–285. 10.1111/nous.12062

[CR42] Itti L, Baldi P (2006) Bayesian surprise attracts human attention. Adv Neural Inf Process Syst 18:547

[CR43] Itti L, Koch C (2000) A saliency-based search mechanism for overt and covert shifts of visual attention. Vision Res 40:1489–1506. 10.1016/S0042-6989(99)00163-7 10788654 10.1016/s0042-6989(99)00163-7

[CR44] Jefferys WH, Berger JO (1992) Ockham’s Razor and Bayesian analysis. Am Sci 80:64–72

[CR45] Kaelbling LP, Littman ML, Cassandra AR (1998) Planning and acting in partially observable stochastic domains. Artif Intell 101:99–134. 10.1016/S0004-3702(98)00023-X

[CR46] Kalman RE & Bucy RS (1961) New results in linear filtering and prediction theory

[CR47] Kaplan R, Friston KJ (2018) Planning and navigation as active inference. Biol Cybern. 10.1007/s00422-018-0753-2 29572721 10.1007/s00422-018-0753-2 PMC6060791

[CR48] Kato S et al (2015) Global brain dynamics embed the motor command sequence of <em>Caenorhabditis elegans</em>. Cell 163:656–669. 10.1016/j.cell.2015.09.034 26478179 10.1016/j.cell.2015.09.034

[CR49] Kiebel S, Daunizeau J, Friston K (2009) Perception and hierarchical dynamics. Front Neuroinformat. 10.3389/neuro.11.020.2009 10.3389/neuro.11.020.2009 PMC2718783 19649171

[CR50] Kinjo K, Uchibe E, Doya K (2013) Evaluation of linearly solvable Markov decision process with dynamic model learning in a mobile robot navigation task. Front Neurorobot 7:7–7. 10.3389/fnbot.2013.00007 23576983 10.3389/fnbot.2013.00007 PMC3617398

[CR51] Knill DC, Pouget A (2004) The Bayesian brain: the role of uncertainty in neural coding and computation. Trends Neurosci 27:712–719 15541511 10.1016/j.tins.2004.10.007

[CR52] Kurby CA, Zacks JM (2008) Segmentation in the perception and memory of events. Trends Cogn Sci 12:72–79. 10.1016/j.tics.2007.11.004 18178125 10.1016/j.tics.2007.11.004 PMC2263140

[CR53] Kuznetsov NV, Mokaev TN, Kuznetsova OA, Kudryashova EV (2020) The Lorenz system: hidden boundary of practical stability and the Lyapunov dimension. Nonlinear Dyn 102:713–732. 10.1007/s11071-020-05856-4

[CR54] LeCun Y, Bengio Y, Hinton G (2015) Deep learning. Nature 521:436–444. 10.1038/nature14539 26017442 10.1038/nature14539

[CR55] Lindley DV (1956) On a measure of the information provided by an experiment. Ann Math Statist 27:986–1005. 10.1214/aoms/1177728069

[CR56] Loeliger HA et al (2007) The factor graph approach to model-based signal processing. Proc IEEE 95:1295–1322. 10.1109/JPROC.2007.896497

[CR57] Lorenz EN (1963) Deterministic nonperiodic flow. J Atmos Sci 20:130–141

[CR58] Louie K, Wilson MA (2001) Temporally structured replay of awake hippocampal ensemble activity during rapid eye movement sleep. Neuron 29:145–156. 10.1016/S0896-6273(01)00186-6 11182087 10.1016/s0896-6273(01)00186-6

[CR59] Maisto D, Gregoretti F, Friston K & Pezzulo G (2021) Active inference tree search in large POMDPs. *arXiv preprint *arXiv:2103.13860.

[CR60] Mannella F, Maggiore F, Baltieri M & Pezzulo G (2021) Active inference through whiskers. *bioRxiv*, 2021.2007.2016.452665, doi:10.1101/2021.07.16.452665. 10.1016/j.neunet.2021.08.037 34563752

[CR61] Marder E, Bucher D (2001) Central pattern generators and the control of rhythmic movements. Curr Biol 11:R986–R996. 10.1016/S0960-9822(01)00581-4 11728329 10.1016/s0960-9822(01)00581-4

[CR62] Miall RC, Weir DJ, Stein JF (1993) Intermittency in human manual tracking tasks. J Mot Behav 25:53–63. 10.1080/00222895.1993.9941639 12730041 10.1080/00222895.1993.9941639

[CR63] Mirza MB, Adams RA, Mathys CD, Friston KJ (2016) Scene construction, visual foraging, and active inference. Front Comput Neurosci. 10.3389/fncom.2016.00056 27378899 10.3389/fncom.2016.00056 PMC4906014

[CR64] Mirza MB, Adams RA, Mathys C, Friston KJ (2018) Human visual exploration reduces uncertainty about the sensed world. PLoS ONE 13:e0190429. 10.1371/journal.pone.0190429 29304087 10.1371/journal.pone.0190429 PMC5755757

[CR65] Morse M, Hedlund GA (1938) Symbolic dynamics. Am J Math 60:815–866. 10.2307/2371264

[CR66] Navas F, Stark L (1968) Sampling or intermittency in hand control system dynamics. Biophys J 8:252–302. 10.1016/S0006-3495(68)86488-4 5639937 10.1016/S0006-3495(68)86488-4 PMC1367375

[CR67] O'Keefe J & Dostrovsky J (1971) The hippocampus as a spatial map: preliminary evidence from unit activity in the freely-moving rat. *Brain research*. 10.1016/0006-8993(71)90358-1 5124915

[CR68] Parr T, Pezzulo G (2021) Understanding, explanation, and active inference. Front Syst Neurosci. 10.3389/fnsys.2021.772641 34803619 10.3389/fnsys.2021.772641 PMC8602880

[CR69] Parr T, Markovic D, Kiebel SJ, Friston KJ (2019) Neuronal message passing using mean-field, Bethe, and marginal approximations. Sci Rep 9:1889. 10.1038/s41598-018-38246-3 30760782 10.1038/s41598-018-38246-3 PMC6374414

[CR70] Parr T, Limanowski J, Rawji V, Friston K (2021) The computational neurology of movement under active inference. Brain. 10.1093/brain/awab085 33704439 10.1093/brain/awab085 PMC8320263

[CR91] Parr T, Pezzulo G, Friston K (2022) Active Inference. The Free Energy Principle in Mind, Brain, and Behavior. MIT Press

[CR71] Penny WD (2012) Comparing dynamic causal models using AIC. BIC Free Energy Neuroimage 59:319–330. 10.1016/j.neuroimage.2011.07.039 21864690 10.1016/j.neuroimage.2011.07.039 PMC3200437

[CR72] Pezzulo G, van der Meer MAA, Lansink CS, Pennartz CMA (2014) Internally generated sequences in learning and executing goal-directed behavior. Trends Cogn Sci 18:647–657. 10.1016/j.tics.2014.06.011 25156191 10.1016/j.tics.2014.06.011

[CR73] Pezzulo G, Kemere C, van der Meer MAA (2017) Internally generated hippocampal sequences as a vantage point to probe future-oriented cognition. Ann N Y Acad Sci 1396:144–165. 10.1111/nyas.13329 28548460 10.1111/nyas.13329

[CR92] Pezzulo G, Rigoli F, Friston K (2018) Hierarchical Active Inference: a Theory of Motivated Control. Trends in Cogn Sci 22(4):294-306 10.1016/j.tics.2018.01.009 PMC5870049 29475638

[CR74] Pio-Lopez L, Nizard A, Friston K, Pezzulo G (2016) Active inference and robot control: a case study. J R Soc Interface 13:20160616. 10.1098/rsif.2016.0616 27683002 10.1098/rsif.2016.0616 PMC5046960

[CR75] Rabinovich M, Bick C & Varona P Beyond neurons and spikes: *cognon*, the dynamical unit of thoughts allowing a theory of spatio-temporal memory and binding. (In preparation).

[CR76] Rao RP, Ballard DH (1999) Predictive coding in the visual cortex: a functional interpretation of some extra-classical receptive-field effects. Nat Neurosci 2:79–87 10195184 10.1038/4580

[CR77] Redish AD (2016) Vicarious trial and error. Nat Rev Neurosci 17:147–159. 10.1038/nrn.2015.30 26891625 10.1038/nrn.2015.30 PMC5029271

[CR78] Schwartenbeck P et al (2019) Computational mechanisms of curiosity and goal-directed exploration. Elife 8:e41703. 10.7554/eLife.41703 31074743 10.7554/eLife.41703 PMC6510535

[CR79] Schwartenbeck P & Friston K (2016) Computational phenotyping in psychiatry: A worked example. *eNeuro* **3**, ENEURO.0049-0016.2016, 10.1523/ENEURO.0049-16.2016 10.1523/ENEURO.0049-16.2016 PMC4969668 27517087

[CR80] Shipp S, Adams RA, Friston KJ (2013) Reflections on agranular architecture: predictive coding in the motor cortex. Trends Neurosci 36:706–716. 10.1016/j.tins.2013.09.004 24157198 10.1016/j.tins.2013.09.004 PMC3858810

[CR81] Srinivasan MV, Laughlin SB, Dubs A, Horridge GA (1982) Predictive coding: a fresh view of inhibition in the retina. Proc Royal Soc London Ser B Biol Sci 216:427–459. 10.1098/rspb.1982.0085 10.1098/rspb.1982.0085 6129637

[CR82] Sutskever I, Vinyals O & Le QV (2014) Sequence to sequence learning with neural networks. *Adv Neural Inform Process Syst*, **27**

[CR83] Sutton RS & Barto AG (1998) *Reinforcement learning: an introduction*. (MIT Press, 1998).

[CR84] Todorov E (2007) Linearly-solvable Markov decision problems. In: Advances in neural information processing systems (vol 19, pp 1369–1376). MIT Press, Cambridge

[CR85] Volterra V (1928) Variations and fluctuations of the number of individuals in animal species living together. ICES J Mar Sci 3:3–51. 10.1093/icesjms/3.1.3

[CR86] Winn J, Bishop CM (2005) Variational message passing. J Mach Learn Res 6:661–694

[CR87] Woodworth RS (1899) Accuracy of voluntary movement. Psychol Rev Monogr Suppl 3:i–114. 10.1037/h0092992

[CR88] Wymbs NF, Bassett DS, Mucha PJ, Porter MA, Grafton ST (2012) Differential recruitment of the sensorimotor putamen and frontoparietal cortex during motor chunking in humans. Neuron 74:936–946. 10.1016/j.neuron.2012.03.038 22681696 10.1016/j.neuron.2012.03.038 PMC3372854

[CR89] Yang SCH, Lengyel M & Wolpert DM (2016) Active sensing in the categorization of visual patterns. *eLife*, **5**, e12215, 10.7554/eLife.12215 10.7554/eLife.12215 PMC4764587 26880546

